# Treatment of displaced transverse fractures of the patella: modified tension band wiring technique with or without augmented circumferential cerclage wire fixation

**DOI:** 10.1186/s12891-018-2092-9

**Published:** 2018-05-24

**Authors:** Tien-Yu Yang, Tsan-Wen Huang, Po-Yao Chuang, Kuo-Chin Huang

**Affiliations:** 1 Chang Gung University College of Medicine, Taoyuan, Taiwan; 2 Department of Orthopaedic Surgery, Chang Gung Memorial Hospital, Chiayi, Taiwan; 3 Putz City, Taiwan

**Keywords:** Patella fracture, Tension band wiring, Circumferential cerclage wiring, Fixation failure, Nonunion

## Abstract

**Background:**

Displaced transverse fractures of the body of the patella are usually associated with disruption of extensor mechanism and should be fixed surgically. The most common method is a tension band wiring (TBW) technique. Some surgeons concurrently employ an augmented circumferential cerclage wiring (ACCW) technique to help fracture stabilization and aid in fracture healing; however, its role and effect on the treatment outcomes is unclear.

**Methods:**

We performed a STROBE-compliant retrospective observational cohort study on all cases of acute closed patella fracture that were treated at our institution between 2006 and 2012. Of 185 episodes, 72 (38.9%) were eligible for this study according to our inclusion/exclusion criteria. We classified these subjects with AO/OTA type 34-C1.1 or 34-C2 lesions into two groups for analyses: fractures treated with modified TBW and ACCW (group 1, *n* = 27) and those treated with modified TBW alone (group 2, *n* = 45). Plain radiographs were used to evaluate radiographic outcomes and the effect of potential risk factors on fixation failure was analyzed by subgroup comparisons.

**Results:**

Our results revealed that there were no significant differences in the rates of fixation failure (*P* = 0.620), nonunion (*P* = 0.620), and revision surgery (*P* = 0.620) between the groups. Although not statistically significant, there was a trend towards a positive risk association between fixation failure and age distribution > 60 years (10.0% vs. 0.0%, *P* = 0.124; OR = 8.0, *P* = 0.168) and > 70 years (9.4% vs. 2.5%, *P* = 0.321; OR = 4.0, *P* = 0.237) and the superficial level of the K-wires (12.0% vs. 1.5%, *P* = 0.117; OR = 6.3, *P* = 0.121). Regarding those modified TBW patients concurrently treated with an ACCW, the potential risk association between fixation failure and the superficial level of the K-wire was prone to increase further (28.6% vs. 0.0%, *P* = 0.060; OR = 18.6, *P* = 0.071).

**Conclusions:**

Concurrent application of an ACCW might be needless and not efficacious to help fracture stabilization and healing in patients having been treated with modified TBW for displaced transverse fractures of the body of the patella. Adherence to correct surgical technique such as putting the K-wires at the proper level and securing control of the both ends of the K-wires may be more important and help in improving outcomes.

## Background

Among all fracture types of the patella, transverse fracture of the body is the most common [1–4]. If displaced, it should be repaired surgically to restore the function of extensor mechanism and aid in fracture healing [5, 6]. Tension band wiring (TBW) technique, one of the most commonly used surgical method in treating a tension fracture [7–10], has a sound biomechanical advantage such that it can convert a tensile force into a compressive force when the joint is brought through a range of motion [11–13]. With appropriate application of this technique to treat the displaced transverse fractures of the patella, the security of fixation would allow early joint mobilization and the prevention of knee stiffness [14, 15]. The rate of fixation failure is not high and serious complications are uncommon [16]. Despite the potential occurrence of subjective complaints due to hardware irritation, TBW remains the gold standard for the treatment of displaced transverse fractures of the patella body [2, 3, 7, 8, 14–17]. At our institution, a modified TBW technique involving bending both ends of the K-wires is used regularly. Using this modified technique, Wu et al. had reported an excellent treatment result with smooth fracture union, good recovery of joint motion, and few complaints of hardware irritation (3%) [15].

Even though the treatment results of TBW for displaced transverse patella fractures are promising, some surgeons recommend that the combination of augmented circumferential cerclage wiring (ACCW) and TBW would lead to a greater strength of fixation and should be better than TBW alone [18–20]. Curtis, in a cadaveric study on the strength of internal fixation for a fractured patella, compared Müller’s TBW technique to the Pyrford technique using a cerclage wire and an anterior TBW looping through the quadriceps tendon [21, 22]. He found that all patellae fixed using Müller’s technique failed at an applied force of 20 kg or less and none of the specimens fixed by the Pyrford technique failed at 25 kg [22]. Accordingly, we proposed a null hypothesis that concurrent application of an ACCW does not help fracture stabilization and enhance fracture healing in patients having been treated by TBW for their displaced transverse fractures of the body of the patella. Through a retrospective cohort study design, we tested the hypothesis based on the data (evidence) collected and then decided to either reject the null hypothesis or failed to reject it. The purpose of this study was (1) to determine the impact of an ACCW on the radiographic outcomes and (2) to explore the prognostic factors predicting treatment failure (e.g., fixation failure) in these patients.

## Methods

### Study population and primary/secondary endpoints

This retrospective cohort study included all patients with a diagnosis of displaced transverse fractures of the patella (AO/OTA type 34-C1.1 and 34-C2) who were treated by modified TBW [15] at our institution between January 2006 and June 2012. After obtaining approval from the Institutional Review Board, cases were identified by matching the International Classification of Diseases (ICD), 9th Revision codes specific for closed patella fracture (822.0) in the computerized trauma registry database of the hospital. At this stage, 185 patients with an acute patella fracture were identified and their medical records were reviewed and confirmed by two independent researchers (TYY and KCH). Among them, we excluded patients aged < 18 years, lost to follow up within 3 months after surgery and those with inadequate radiographs, bilateral patella fractures, open or multiple fractures, vertical fractures, complex fractures, and avulsion fractures of the upper or lower pole of the patella. The aggregate number of enrolled patients in this 6.5-year study was 72.

We summarized the collected data at the time of study enrollment, which included demographics (age and gender), AO/OTA fracture types, surgical methods, aftercare program, radiographic results and surgical complications. To clarify the role of an ACCW on treatment outcomes of displaced transverse patella fracture, we classified the enrolled subjects into two groups for further analyses: fractures treated with modified TBW and ACCW (group 1, *n* = 27) and those with modified TBW alone (group 2, *n* = 45). The primary endpoint of this study was to determine the impact of an ACCW on the radiographic results of displaced transverse patella fracture having been treated by modified TBW. For our secondary endpoint, the effect of potential risk factors on fixation failure was analyzed by subgroup comparisons.

### Definitions

#### Level of the K-wires

It was categorized as superficial or deep. Superficial level was defined as the level of the K-wires anterior to the center of the patella. In contrast, we defined the deep level as being in the center of deeper position of the patella.

#### Distance between the K-wires

It was categorized as narrow or wide. Narrow distance was defined as the distance between the K-wires less than one third of the patella width. In contrast, we defined the wide distance as being equal or more than one third of the patella width.

#### Fixation failure

It was defined as hardware breakage, nonunion, or redisplacement of fragments from their initial reduced position with gap separation > 2 mm.

### Statistical analysis

Variables were compared using univariate analysis. A χ^2^ analysis or a Fisher’s exact test was used where appropriate for analyzing categorical data. For numerical data, the Wilcoxan rank sum test was utilized for between-group comparisons. Significance was set at *P* < 0.05 (two-sided). Not statistically significant was abbreviated as NS. All statistic analyses were conducted using the Statistical Package for the Social Sciences (SPSS, version 12.0. SPSS Inc., Chicago, IL, USA) and/or the MedCalc Statistical Software (version 9.5, Broekstraat, Mariakerke, Belgium).

## Results

According to the inclusion/exclusion criteria, 72 (38.9%) of 185 episodes were eligible for this study and divided into two groups, including the group of fractures treated with modified TBW and ACCW (group 1, *n* = 27) and the group of those treated with modified TBW alone (group 2, *n* = 45). Baseline data on these 72 patients treated with modified TBW for displaced transverse fractures of the patella are shown in Table 1. Age, age distribution, gender, fracture pattern, level of the K-wires, distance between the K-wires, and aftercare such as use of postoperative splint did not differ between the two groups of patients. Regarding the outcome analysis, there were no significant differences in the rates of fixation failure (*P* = 0.620), nonunion (*P* = 0.620), or revision surgery (*P* = 0.620) between the groups (Table 2). In total there were 4 patients (5.6%) having fixation failure, including 2 group-1 patients (7.4%) and 2 group-2 patients (4.4%), respectively. All of them had nonunion but no wire migration and needed revision surgery to restore their extensor mechanism and enhance fracture union. After bone grafting and revision modified TBW surgery focusing on the adjustment of the level of the K-wires and the distance between them, all these fixation failure patients recovered their function of extensor mechanism and had their patella fractures united eventually (Table 3).

**Table 1:** Characteristics of patients treated by tension band wiring technique with or without augmented cerclage wire fixation

Variables	Group 1 (*n* = 27)	Group 2 (*n* = 45)	*P* Value
Age (years), mean (range)	61.33 (32–87)	63.73 (25–88)	0.530
Age > 60 years, n (%)			0.341
Yes	13 (48.1)	27 (60.0)	
No	14 (51.9)	18 (40.0)	
Age > 70 years, n (%)			0.807
Yes	11 (40.7)	21 (46.7)	
No	16 (59.3)	24 (53.3)	
Gender, n (%)			0.802
Male	11 (40.7)	16 (35.6)	
Female	16 (59.3)	29 (64.4)	
Fracture pattern, n (%)			0.535
AO/OTA type 34-C2	6 (22.2)	7 (15.6)	
AO/OTA type 34-C1.1	21 (77.8)	38 (84.4)	
Level of the K-wires, n (%)			0.308
Superficial	7 (25.9)	18 (40.0)	
Deep	20 (74.1)	27 (60.0)	
Distance between the K-wires, n (%)			0.085
Narrow	12 (44.4)	30 (66.7)	
Wide	15 (55.6)	15 (33.3)	
Postoperative splinting, n (%)			0.807
Yes	16 (59.3)	24 (53.3)	
No	11 (40.7)	21 (46.7)	

*P value < 0.05 is significant

**Table 2:** Radiographic results and revision surgery of patients treated by tension band wiring technique with or without augmented cerclage wire fixation

Variables	Group 1 (*n* = 27)	Group 2 (*n* = 45)	*P* Value
Fixation failure, n (%)			0.620
Yes	2 (7.4)	2 (4.4)	
No	25 (92.6)	43 (95.6)	
Nonunion, n (%)			0.620
Yes	2 (7.4)	2 (4.4)	
No	25 (92.6)	43 (95.6)	
Wire migration, n (%)			1.000
Yes	0 (0.0)	0 (0.0)	
No	27 (100.0)	45 (100.0)	
Revision surgery, n (%)			0.620
Yes	2 (7.4)	2 (4.4)	
No	25 (92.6)	43 (95.6)	

*P value < 0.05 is significant

**Table 3:** Demographic data of the cases with fixation failure

Variables	Group 1		Group 2	
	Patient 1	Patient 2	Patient 3	Patient 4
Age, years	66	78	73	88
Gender	F	M	F	M
AO/OTA type 34-C2 fracture	N	N	N	N
Superficial level of the K-wires	Y	Y	N	Y
Narrow K-wire distance	Y	N	Y	N
Without postoperative splinting	Y	N	Y	N
K-wire migration	N	N	N	N
Revision surgery	Y	Y	Y	Y
Final union	Y	Y	Y	Y

*P value < 0.05 is significant

Several potential risk factors for fixation failure were analyzed and shown in Table 4. Although not statistically significant, we observed a trend of positive risk association between fixation failure and age distribution > 60 years (10.0% vs. 0.0%, *P* = 0.124; OR = 8.0, *P* = 0.168) and > 70 years (9.4% vs. 2.5%, *P* = 0.321; OR = 4.0, *P* = 0.237) and the superficial level of the K-wires (12.0% vs. 2.1%, *P* = 0.117; OR = 6.3, *P* = 0.121). Regarding those patients concurrently treated with an ACCW, the risk association between fixation failure and the superficial level of the K-wire increased further (28.6% vs. 0.0%, *P* = 0.060; OR = 18.6, *P* = 0.071).

**Table 4:** Prognostic factor analyses: subgroup comparisons of the effects of patient characteristics on fixation failure

Variables	Fixation Failure	Union	*P* Value	OR (95% CI)	*P* Value
All patients, n (%)	(*n* = 4)	(*n* = 68)			
Patient age > 60 years	4 (100.0)	36 (52.9)	0.124	8.0 (0.4–154.6)	0.168
Patient age > 70 years	3 (75.0)	29 (42.6)	0.321	4.0 (0.4–40.8)	0.237
Female patients	2 (50.0)	43 (63.2)	0.628	0.6 (0.1–4.4)	0.599
AO/OTA type 34 C2 fracture	0 (0.0)	13 (19.1)	1.000	0.5 (0.0–9.0)	0.607
Superficial level of the K-wires	3 (75.0)	22 (32.3)	0.117	6.3 (0.6–63.8)	0.121
Narrow K-wire distance	2 (50.0)	40 (58.8)	1.000	0.7 (0.1–5.3)	0.729
Without postoperative splinting	2 (50.0)	30 (44.1)	1.000	1.3 (0.2–9.5)	0.818
Group 1 patients, n (%)	(*n* = 2)	(*n* = 25)			
Patient age > 60 years	2 (100.0)	11 (44.0)	0.222	6.3 (0.3–144.7)	0.249
Patient age > 70 years	1 (50.0)	10 (40.0)	1.000	1.5 (0.1–26.9)	0.783
Female patients	1 (50.0)	15 (60.0)	1.000	0.1 (0.0–3.1)	0.212
AO/OTA type 34 C2 fracture	0 (0.0)	6 (24.0)	1.000	0.6 (0.0–14.2)	0.752
Superficial level of the K-wires	2 (100.0)	5 (20.0)	0.060	18.6 (0.8–447.7)	0.071
Narrow K-wire distance	1 (50.0)	11 (44.0)	1.000	1.3 (0.1–22.7)	0.870
Without postoperative splinting	1 (50.0)	10 (40.0)	1.000	1.5 (0.1–26.9)	0.783
Group 2 patients, n (%)	(*n* = 2)	(*n* = 43)			
Patient age > 60 years	2 (100.0)	25 (58.1)	0.509	3.6 (0.2–80.1)	0.415
Patient age > 70 years	2 (100.0)	19 (44.2)	0.212	6.3 (0.3–138.6)	0.244
Female patients	1 (50.0)	28 (65.1)	1.000	0.5 (0.0–9.2)	0.667
AO/OTA type 34 C2 fracture	0 (0.0)	7 (16.3)	1.000	1.0 (0.0–22.4)	0.987
Superficial level of the K-wires	1 (50.0)	17 (39.5)	1.000	1.5 (0.1–26.1)	0.769
Narrow K-wire distance	1 (50.0)	29 (67.4)	1.000	0.5 (0.0–8.3)	0.616
Without postoperative splinting	1 (50.0)	20 (66.7)	1.000	1.2 (0.1–19.6)	0.923

*P value < 0.05 is significant

## Discussion

The decision of the current retrospective cohort study was failure to reject the null hypothesis [23], i.e., there was no sufficient evidence supporting that the concurrent application of an ACCW could provide a better treatment outcome (e.g., less fixation failure) than modified TBW alone while treating patients with displaced transverse fractures of the patella. The fixation failure rate in group 1 and group 2 patients was 7.4 and 4.4%, respectively (*P* = NS). All these fixation failure patients recovered their function of extensor mechanism and had their patella fractures united by bone grafting and revision surgery with modified TBW alone, focusing on the adjustment of the level of the K-wires and the distance between them. Although not statistically significant, prognostic factor analyses showed that there was a trend towards a higher rate of fixation failure in elderly patients (OR ≥ 4.0, *P* = NS) and those with their K-wires at the superficial level (OR = 6.3, *P* = NS). Concurrently applying an ACCW did not decrease the risk association between fixation failure and the superficial level of the K-wires (OR = 18.6 and 1.5, both *P* = NS, in group 1 and group 2 patients, respectively). We, thus, highlight that concurrent application of an ACCW in patients having been treated with modified TBW technique might be not as efficacious as what we had learned from the literature [18–20]. Reviewing the literature showed that all of these clinical articles were categorized as case-series, a study design lacking comparison groups and thus vulnerable to selection bias and easily leading us to an incorrect conclusion [24, 25]. To further clarify the role and effect of concurrent application of an ACCW on the treatment outcomes, a double-blinded prospective randomized controlled trial is, therefore, necessary in the future.

To the best of our knowledge, this is the first report to determine the impact of concurrently applying an ACCW on the treatment outcomes of displaced transverse patella fractures having been fixed by modified TBW. The present study also provides a higher level of evidence than that of previously published articles by others [18–20]. Moreover, our results challenge the concept proposed and propelled by Curtis in 1990 [22]. After comparing Pyrford technique (TBW + ACCW) to Müller’s technique (TBW alone) in a cadaveric study, Curtis concluded that the former gave greater strength of fixation and should be recommended. However, nearly all patellae fixed using Müller’s TBW technique failed when the distal ends of the K-wire bent, which is also true in clinical scenario [26–29]. Because of the difficulty capturing the tension band by the migrated or bent distal ends of the K-wires, loss of fixation or reduction can occur in as many as 20% of patella fractures treated with Müller’s TBW technique [26–29]. Many revised techniques and technologies were, therefore, developed to solve this problem [15, 18–20, 27–30]. Wu’s modified TBW technique is one of the simplest and most effective methods [15]. Using this technique in this study, the total fixation failure rate was 5.6% only. It’s interesting that concurrently applying an ACCW could not provide a superior result than Wu’s modified TBW alone. Considering the potential hazards on patella vascularity and the non-superiority results, we, therefore, recommend that concurrent application of an ACCW may not be required. Adherence to correct surgical technique of modified TBW technique might be more important and help in improving outcomes.

Our results suggested that the level of the K-wires might be one of the most critical surgical pearls in the treatment of patients with displaced transverse fractures of the body of the patella. The TBW design for treating these lesions needs to handle compression and tension without buckling and snapping. When the compression force overcomes bone’s ability to handle compression, buckling occur [31]. When the tension force overcomes wires’ ability to handle tension, snapping occurs [26–29, 32–34]. The TBW technique should transfer and dissipate these forces. Putting the parallel K-wires at the superficial level (i.e., anterior to the center of the patella) and with a narrow distance between them (i.e., less then one third of the patellar width) might be inadequate to transfer and dissipate the compression force, thus resulting in buckling and cut-through phenomena [32]. Applying the TBW without securing control of the both ends of the K-wires might be inadequate to transfer and dissipate the tension force, thus resulting in snapping and wire migration, bending, or breakage [33, 34]. Our findings indicated that Wu’s modified TBW technique could effectively control the both ends of the K-wires, thus eliminate the possibility of wire migration/bending/breakage and then improve the fixation stability. Concurrently applying an ACCW could not further help the current fixation design handle compression and tension forces. Instead, putting the K-wires at the deep level and with wide distance between them might be more helpful for handling these deforming forces.

Osteoporotic bone is at risk for fractures and fixation failure after surgical stabilization [35]. To determine anatomic sites and circumstances under which a fracture may be a consequence of osteoporosis, Warriner et al. developed the osteoporosis attribution scoring (OAS) system and provided an evidence-based continuum of the likelihood of a fracture being associated with osteoporosis. Using the OAS system they categorized patella fracture as a high likelihood of being because of osteoporosis in older Caucasian women but an indeterminate likelihood in younger African-American man [36]. Our results revealed that there was a paradoxical relationship between fixation failure in patella fractures and some osteoporosis risk factors (e.g., aging, gender and bone quality) [37, 38]. We found a trend towards an increased risk of fixation failure in geriatric patients; however, the risk association did not increase with increasing age. Female gender, fracture comminution (AO/OTA type 34-C2) and without use of postoperative splint were not associated with an increased risk of fixation failure. Whatever role osteoporosis might play in the treatment of patella fractures, we believe that refinement of TBW technique and/or technology to successfully handle the deforming forces should be the most critical surgical pearls. The refined technique and/or technology would effectively improve fixation stability, permit early joint motion, enhance fracture healing, promise an excellent treatment outcome, and thus decrease the cost burden on the family and society for that condition.

Our study has several limitations. First, it is retrospective. Second, the number of patients with treatment failure included during the study period was relatively small and the study may have lacked power to detect the statistical differences in all prognostic factors among subsets of patients. The uncommon occurrence of fixation failure in the current study is to be expected because of the excellent results provided with Wu’s modified TBW technique [15]. After a consecutive follow-up study lasting for more than 6 years, our results highlighted the importance of adherence to correct modified TBW technique instead of adding an ACCW in treating these patients. Third, there is lack of functional outcome measures in this study. For treatment of displaced transverse fractures of the patella, TBW technique has been a well-documented surgical method. The role of concurrently applying an ACCW to fractured patella having been fixed with TBW is intended to help fracture stabilization. The purpose of this study was therefore to determine the impact of concurrent application of an ACCW on the treatment outcomes (i.e., fixation failure) until bone healing, leaving functional outcome consideration aside. Lastly, only AO/OTA type 34-C1.1 or 34-C2 fractures were included in this investigation. We excluded all complex fractures and avulsion fractures of the proximal or distal pole of the patella in the current work in order to reduce systemic errors and bias to the smallest possible degree.

## Conclusion

We concluded that there was no sufficient evidence supporting that the concurrent application of an ACCW could provide a better treatment outcome (e.g., less fixation failure) than Wu’s modified TBW alone while treating patients with a displaced transverse fracture of the patella. Wu’s modified TBW technique could effectively control the both ends of the K-wires, thus eliminate the possibility of wire migration/bending/breakage and then improve the fixation stability. Putting the K-wires at the deep level and with wide distance between them might effectively transfer and dissipate the compression force, thus decrease the chance of cut-through phenomena and loss of fixation/reduction. Considering the potential hazards on patella vascularity and the non-superiority results, we, therefore, recommend that concurrent application of an ACCW may not be required.

## Abbreviations

ACCW: Augmented circumferential cerclage wire
AO/OTA: Arbeitsgemeinschaft für Osteosynthesefragen/Orthopaedic Trauma Association
ICD: International Classification of Diseases
K-wire: Kirschner-wire
NS: not significant
OAS: Osteoporosis Attribution Scoring
OR: odds ratio
SPSS: Statistical Product and Service Solutions
STROBE: Strengthening the Reporting of Observational studies in Epidemiology
TBW: tension band wire
